# De novo transcriptomic analysis of hydrogen production in the green alga *Chlamydomonas moewusii* through RNA-Seq

**DOI:** 10.1186/1754-6834-6-118

**Published:** 2013-08-23

**Authors:** Shihui Yang, Michael T Guarnieri, Sharon Smolinski, Maria Ghirardi, Philip T Pienkos

**Affiliations:** 1 National Bioenergy Center, Golden, CO, USA; 2 Biosciences Center, National Renewable Energy Laboratory, Golden, CO 80401, USA

## Abstract

**Background:**

Microalgae can make a significant contribution towards meeting global renewable energy needs in both carbon-based and hydrogen (H_2_) biofuel. The development of energy-related products from algae could be accelerated with improvements in systems biology tools, and recent advances in sequencing technology provide a platform for enhanced transcriptomic analyses. However, these techniques are still heavily reliant upon available genomic sequence data. *Chlamydomonas moewusii* is a unicellular green alga capable of evolving molecular H_2_ under both dark and light anaerobic conditions, and has high hydrogenase activity that can be rapidly induced. However, to date, there is no systematic investigation of transcriptomic profiling during induction of H_2_ photoproduction in this organism.

**Results:**

In this work, RNA-Seq was applied to investigate transcriptomic profiles during the dark anaerobic induction of H_2_ photoproduction. 156 million reads generated from 7 samples were then used for de novo assembly after data trimming. BlastX results against NCBI database and Blast2GO results were used to interpret the functions of the assembled 34,136 contigs, which were then used as the reference contigs for RNA-Seq analysis. Our results indicated that more contigs were differentially expressed during the period of early and higher H_2_ photoproduction, and fewer contigs were differentially expressed when H_2_-photoproduction rates decreased. In addition, *C. moewusii* and *C. reinhardtii* share core functional pathways, and transcripts for H_2_ photoproduction and anaerobic metabolite production were identified in both organisms. *C. moewusii* also possesses similar metabolic flexibility as *C*. *reinhardtii*, and the difference between *C*. *moewusii* and *C*. *reinhardtii* on hydrogenase expression and anaerobic fermentative pathways involved in redox balancing may explain their different profiles of hydrogenase activity and secreted anaerobic metabolites.

**Conclusions:**

Herein, we have described a workflow using commercial software to analyze RNA-Seq data without reference genome sequence information, which can be applied to other unsequenced microorganisms. This study provided biological insights into the anaerobic fermentation and H_2_ photoproduction of *C*. *moewusii*, and the first transcriptomic RNA-Seq dataset of *C*. *moewusii* generated in this study also offer baseline data for further investigation (e.g. regulatory proteins related to fermentative pathway discussed in this study) of this organism as a H_2_-photoproduction strain.

## Background

Development of renewable energy sources is crucial for energy security, climate change mitigation, and economic recovery. It would be desirable to develop various renewable energy sources such as wind, solar, and geothermal energy, as well as cellulosic biomass-based biofuels and biological H_2,_ to meet the goal of replacing petroleum-based fuel. One approach for capture of solar energy is the exploitation of microalgae, which can photosynthetically produce H_2_ from water, ferment starch into H_2_ and organic acids, and convert CO_2_ into liquid biofuel, providing different renewable energy forms and concurrently contributing to solving the greenhouse gas problem [1-4]. The recently revived interest in algal research has witnessed the accumulation of large algae strain collections and knowledge in strain characterization. In addition, genetics tools for various algae have been developed, and more accurate technoeconomic analyses and downstream processing improvement have also been published recently [5-7]. With recent advances in next-generation sequencing (NGS) and synthetic biology technologies, more progress can be foreseen in the future for algae-based renewable energy [8].

*Chlamydomonas moewusii* is a unicellular green alga capable of evolving molecular H_2_ under both dark and light anaerobic conditions. Meuser et al. (2009) previously compared hydrogenase activity, starch catabolism, and secreted anaerobic metabolites of *Lobochlamys culleus* (syn. *C*. *reinhardtii* UTEX 1060 (mt-)) and four *C*. *moewusii* strains (UTEX 10 (mt^−^), UTEX 97 (mt^+^), UTEX 2018 (mt^−^), and SAG 24.91 (mt^−^)) with the model green alga, *C*. *reinhardtii*. The results indicated that (**i**) *C*. *moewusii* strains had the most rapid H_2_-photoproduction activity induction, followed by faster loss of activity over longer periods of time; (**ii**) the SAG 24.91 strain had the highest in vitro hydrogenase activity of all strains examined, as well as faster rates of starch catabolism; (**iii**) although SAG 24.91 had a rate of dark, fermentative H_*2*_-production activity similar to that of *C*. *reinhardtii* at earlier times, it and all other *C*. *moewusii* strains lost their dark, fermentative H_2_-production activity faster than *C*. *reinhardtii* over time. Finally, *C*. *moewusii* strains had different profiles of secreted anaerobic metabolites [9] when compared to *C*. *reinhardtii*[10]. However, with the exception of nearly 160 protein sequences available at NCBI database, including those from the 23-kb mitochondria genome (NC_001872) [11,12], the complete nuclear and chloroplast genome sequences of *C*. *moewusii* are not available. Furthermore, although systems biology approaches of transcriptomics, metabolomics, and proteomics have been applied for the model green alga *C*. *reinhardtii*[10,13-25], systematic investigation in *C*. *moewusii* has not yet been carried out.

The development of energy-related products from algae could be accelerated with improvements in systems biology tools. Advances in sequencing technology provide a platform for the development of novel transcriptome analysis approaches although most of these techniques are still heavily reliant upon available genomic sequence, and only seven genomic sequences are available for green microalgae species of *Ostreococcus tauri*, *C*. *reinhardtii*, *Micromonas sp*., *Chlorella variabilis*, *Volvox carteri*, *Coccomyxa subellipsoidea*, and *Ostreococcus lucimarinus*[26-33]. Recently, quite a few studies have applied NGS-based transcriptomics to species without reference genome sequence, such as the studies to identify and construct lipid and starch biosynthesis and catabolism pathways in the microalga *Dunaliella tertiolecta*[34], to discover gene and reconstruct metabolic network for terpenoid biosynthesis in the hydrocarbon oil-producing green alga *Botryococcus braunii* race B [35], to investigate the triacylglyceride (TAG) accumulation mechanism of the unsequenced oleaginous microalgae of *Neochloris oleoabundans* and *Chlorella vulgaris*[36,37], and to unravel the putative mechanisms associated with the successful coastal ecosystems colonization by *Ulva linza*[38]. This powerful technique has not yet been applied to a potential H_2_ production green algal strain without a reference genome.

In this study, we combine commercial software for NGS data analysis and statistical analysis to examine *C*. *moewusii* transcripts responsive to dark anaerobic induction of H_2_ photoevolution through a time-course transcriptomic study. Our work supports the metabolite difference observed previously between *C*. *moewusii* and *C*. *reinhardtii* at transcriptional level, provides insight into the different anaerobic fermentation and H_2_ photoproduction patterns observed in *C*. *moewusii* and *C*. *reinhardtii*, and establishes baseline data for future metabolic engineering and modeling using *C*. *moewusii*. In addition, the transcriptomic data and the contigs generated through this study can facilitate comparative transcriptomics studies among different algae.

## Results and discussions

### Hydrogen evolution and RNA-Seq study

**Figure 1:**
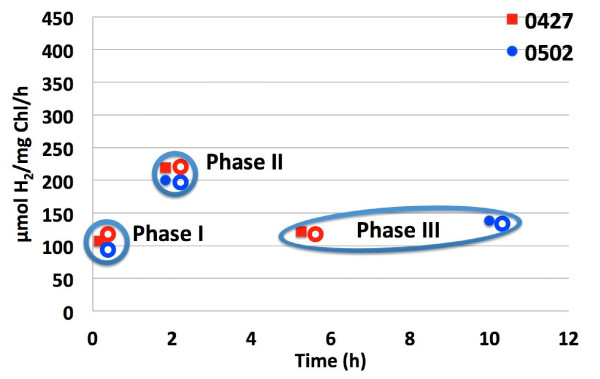
**Hydrogen photoproduction rates of *C. moewusii *sampled at different time points following anaerobic induction.** Two independent experiments (0427 and 0502) are shown here. The closed rectangles and circles indicate the time points at which samples were collected for H2 photoproduction measurement. The open rectangles and circles indicate the sampling time points for total RNA extraction (ca 5 min after the H2 photoproduction points).

Three experiments were carried out to gather H_2_-photoproduction data and collect the cells for total RNA extraction immediately after H_2_ production was measured. In the first experiment, cells were harvested from the aerobic culture prior to anaerobic induction (zero time point) [sample ID: 0 M-0508]. In another two experiments, cells were harvested at four different time-points, at approximately 15 min [15 M-0427 and 15 M-0502], 2 h [2 h-0427 and 2 h-0502], 5 h [5 h-0427], and 10 h [10 h-0502] (Figure 1) following initiation of anaerobic induction.

Together, seven samples were used for total RNA extraction and RNA-Seq using Illumina GAIIx. RNA-Seq data from 0 M-0508 were used for de novo assembly to generate reference contigs only. Three time phases with biological replicate data were used for statistical analysis to understand the transcriptomic profiling during induction of H_2_ photoproduction: 15 M-0427 and 15 M-0502 used as the time point for early H_2_ photoproduction (Phase I); 2 h-0427 and 2 h-0502 used as the time point for high H_2_-photoproduction rates (Phase II); and 5 h-0427and 10 h-0502 used as the time point for decreased rates of H_2_ photoproduction (Phase III) (Figure 1). The gene expression level was indicated by the log_2_-transformed RPKM (Reads Per Kilobase per Million mapped reads) value. For the purpose of clarity, RPKM mentioned throughout the paper refers to the log_2_-transformed RPKM unless otherwise noted.

### Functional annotation of reference contigs generated through de novo assembly for RNA-Seq

The RNA-Seq data analysis flowchart used in this study is illustrated in Additional file 1, which also includes upstream cell culturing and harvest and downstream data interpretation steps. Together, 156 million reads were generated from 7 RNA-Seq samples (Additional file 2). Except for one sample sequence result (15 M-0502) that was provided as trimmed fastq dataset by the sequencing facility with an average length of 36-bp, other RNA-Seq data were provided as raw untrimmed datasets with an average sequence read length of 54-bp. The data quality was checked using FastQC software and the bases with quality score less than 28 were filtered, which left only the high quality reads for the downstream assembly (Additional file 3). As another means of quality control, we adjusted the data using the duplicate reads removal tool of the CLC Genomics Workbench because duplicate reads within the dataset may affect de novo assembly results and misrepresent the real reference contigs generated through assembly. The sequence duplication level did not change after data trimming although the sequence length was shortened. After duplicate reads were removed, the sequence duplication level dropped by more than one-third (from ca 70% duplication to around 44%), but the average sequence length was still close to that before duplicate reads removal (Additional file 2). These results indicate that the data trimming and duplicate removal didn’t change the NGS dataset profiles unexpectedly, and the reads distribution within trimmed data with replicates removed should be representative of the transcriptional profiles of the original datasets. Trimmed raw data with duplicate reads removed were used only for assembly to generate the reference contigs, and trimmed raw data were used for RNA-Seq analyses.

To confirm this and compare the results using different datasets from same seven RNA-Seq data before and after data processing, three datasets (the raw data, trimmed raw data, and trimmed raw data with duplicate reads removed) were then separately used for de novo assembly (Additional file 1). Although most of the parameters (e.g. N50, Maximum length, Average length, Contig number and Total length) of the assembly result using raw data were the largest among the three assembly results (Table 1), the results may contain inaccurate sequences due to the existence of poor quality bases in the raw data (Additional file 3). Comparing the other two assembly results, we saw no dramatic differences (Table 1). However, the assembly using the trimmed raw data with duplicate reads removed generated the largest total length and greatest number of contigs, as well as a larger maximum length. In addition, the lengths of nearly all the contigs contribute to the total length (Additional file 4A). The 34,136 contigs generated from this assembly result were therefore used as the reference contigs for further RNA-Seq analysis (Table 1, Additional file 5A). Approximately three quarters of the contigs were less than 1-kb and the average length is approximately 716-bp, with a range from a minimum length of 194-bp to a maximum length of 15,688-bp (Additional file 4B). Although contigs with short length (e.g. 9,722 contigs with length less than 300-bp) may not be real transcripts, we decided to keep them to avoid complications with false negatives, accepting that the incidence of false positives could increase. In addition, while contigs generated through our assembly have high quality for use as references for RNA-Seq analysis, as discussed below on the three key points of contiguity, completeness, and correctness in assessing assembly quality, recent improvements in NGS technology can provide even better contig connectivity with longer reads length for better assembly than those applied in this study.

**Table 1:** **Determine the reference transcripts through de novo assemblies using different sets of data**

**Contig measurements**	**Raw data**	**Trimmed data**	**Trimmed data with duplicates removed**
N75	555	524	517
N50	1,160	1,063	1,066
N25	1,998	1,829	1,846
Minimum length (bp)	183	196	194
Maximum length (bp)	15,688	14,561	15,688
Average length (bp)	758	718	716
Contig number	39,431	33,374	34,136
Total length (Mb)	29.9	24.0	24.4

**Raw Data**: original data generated from Illumina RNA-Seq; **Trimmed Data**: original data processed to trim the reads with poor quality using CLC Genomics software based on quality analysis result using FastQC software; **Trimmed Data with Duplicates Removed**: the trimmed reads with duplicate reads removed using CLC Genomics software.

**Figure 2:**
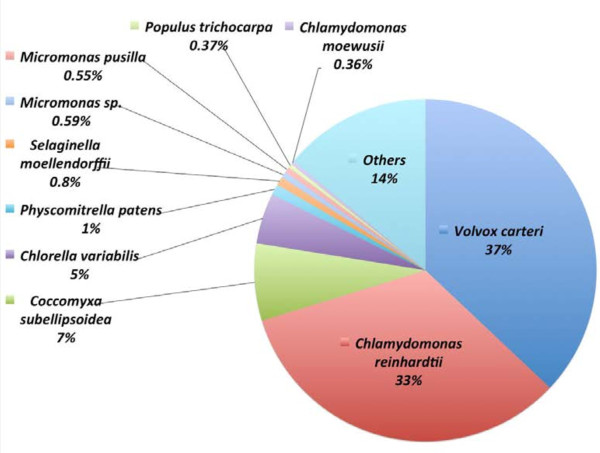
**BlastX results showing Top-Hit species distribution for the 34,136 *C*. *moewusii *transcripts based on Blast2GO result.**

BlastX and Blast2GO programs [39-41] were then applied to the FASTA data of the 34,136 contigs generated from the de novo assembly in order to obtain their functional assignments (Additional file 1). The BlastX search had positive hits for nearly all the contigs, with 2,000 contigs related to ribosomal proteins and 1,700 belonging to transcriptional factors; more than one-third (13,920) are hypothetical proteins with unknown function (Additional file 5A). Nearly all of the top hits with the best BlastX E-values come from other algae, accounting for more than four-fifth of the total contigs (Figure 2). In addition, the high similarity of assembled contigs to 40 *C*. *moewusii* mitochondrial genes (NC_001872) available in NCBI database (Additional file 5B) further supports the use of the annotated assembled contigs as reference for the following time-course transcriptomic study. For example, 30 contigs match the *C*. *moewusii* mitochondrial genome sequence (Additional file 6A). In addition, the longest assembled contig (Contig_490, 15688-bp) matches the flagellar outer dynein arm heavy chain gamma of *V*. *carteri* with 81% identity and 91% similarity (Additional file 6B). Several other long contigs also match to the dynein heavy chain and flagellar components: contig_1136 (8745-bp) matches the dynein heavy chain 6 of *C*. *reinhardtii* with 78% identity and 88% similarity (Additional file 6C); contig_665 (8079-bp) matches the dynein heavy chain beta of *V*. *carteri* with 83% identity and 92% similarity; contig_2517 (8079-bp) matches dynein heavy chain 9 of the same organism with 79% identity and 89% similarity; and contig_649 (9293-bp) matches flagellar-associated callose synthase-like protein of *C*. *reinhardtii* with 62% identity and 78% similarity (Additional file 5A). The high abundance of these flagellar proteins based on RPKM value (ranging from top 25% to 10%) and reads number (ranging from top 10% to 2.5%), as well as the existence of many contigs (>440) related to dynein, also indicate the existence and importance of the flagellar system in *C*. *moewusii* (Additional file 5A, 5C).

Additionally, Blast2Go was employed to identify functional categories of these contigs. In total, 37,781 Gene Ontology (GO) terms were assigned to 10,356 contigs that have at least one GO function and more than 14,000 contigs have InterPro hits (Additional file 5A), of which 6,300 involve in biological processes, 7,851 have molecular function, and 4,851 are structural components (Additional file 5D, 7A-C). In addition, 3,129 enzyme codes were assigned to 2,624 contigs (Additional file 5A) matching 123 KEGG pathways. All enzymes could be placed into specific pathways, such as the one-carbon pool maintained by the folate pathway (map00670) and the C4-dicarboxylic acid cycle for carbon fixation in photosynthetic organisms (map00710) (Additional file 7D, E). These observations lend further support to the usage of assembled contigs as reference for the RNA-Seq study.

### Time-course transcriptomic profiling during dark induction of H_2_ photoproduction

The RPKM values calculated individually for all the 34,136 contigs at each time point from seven RNA-Seq datasets after data trimming were imported into JMP Genomics for statistical analyses (Additional file 5C). The data quality of log_2_-transformed RPKM values for these 7 RNA-Seq datasets was checked using parallel plot and heat-map dendrogram. The results indicated that the RNA-Seq data were of high quality and evenly distributed (Additional file 8). Except for the dataset at 0 min with larger average RPKM values (discussed below), all other datasets had similar distribution patterns (Additional file 8A). Furthermore, the datasets were grouped by different time-points into 4 clusters from all the 3 experiments performed at different times, and the Phase I, II, and III datasets were clustered together as originally grouped based on time points as mentioned above (Additional file 8B).

The RNA-Seq dataset at 0 min was excluded for further statistical analyses due to the lack of a biological replicate and disproportionately larger RPKM values than other datasets (Additional file 8A). The parallel plot of these six RNA-Seq datasets after Loess normalization had a similar distribution pattern (Additional file 8C), and the correlation between the biological replicates was also very tight, with correlation coefficient values of 0.90, 0.91 and 0.87 for Phase I, II, III samples, respectively (Additional file 8D-F). Three comparisons were investigated: Phase I/Phase II from early H_2_-photoproduction induction to peak H_2_-photoproduction rate; Phase II/Phase III from peak H_2_ photoproduction to a late time point with decreased H_2_-photoproduction rate; and Phase I/Phase III for the transcriptomic profile from early H_2_ photoproduction to the end stage, when H_2_-photoproduction rate decreased from peak (Figure 1). For purposes of clarity, all the upregulated or downregulated contigs mentioned below have statistically significant expression (false discovery rate (FDR) <0.05) with a differential expression ratio of at least 2-fold unless otherwise mentioned.

**Figure 3:**
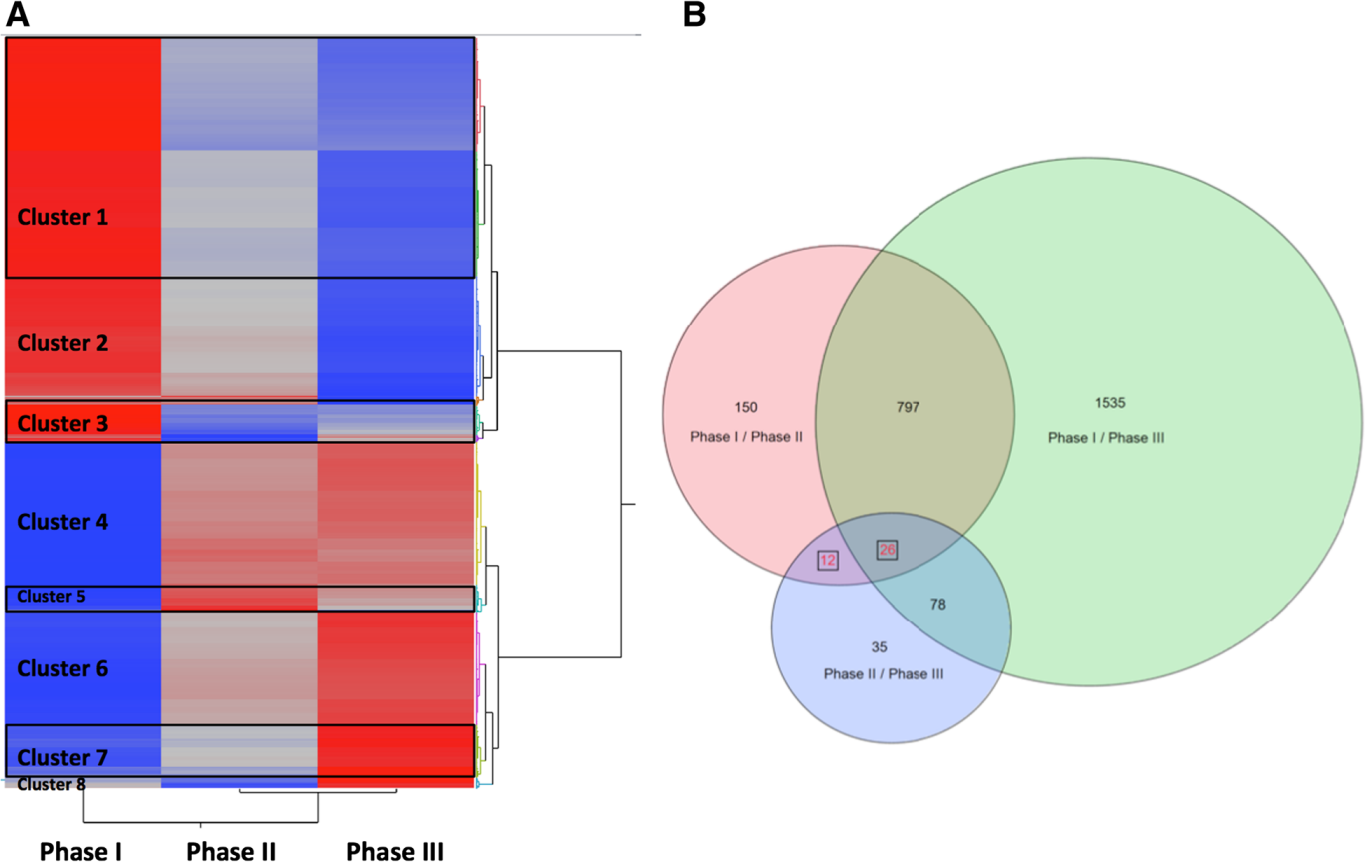
**Clustering analysis of 2, 633 differentially expressed contigs. (A)** Hierarchical clustering of 2,633 differentially expressed transcript abundance in Phases I, II and III of H2 photoproduction; and **(B)** Venn Diagram of the distribution of differentially expressed transcripts among the three different phases of H2 photoproduction.

Although software packages for RNA-Seq data analysis continue to improve, there is no widely-accepted approach yet, especially for datasets with small sample size [42]. The statistical approach we used previously for microarray data analysis for different microorganisms, which has high correlation with qPCR results, was therefore applied in this study [43-45]. A similar approach, using the robust LOWESS normalization was applied in a recent work with good correlation between RNA-Seq and microarray approaches [24]. One-Way ANOVA analysis, using JMP Genomics indicated that 2,633 contigs were significantly differentially-expressed in at least one comparison (Additional file 9A). When the RPKM values of these 2,633 contigs were grouped by Hierarchal Clustering, the transcriptomic profiles in Phase II and Phase III are closer than that of in Phase I, and clustered together (Figure 3A) with more contigs significantly differentially-expressed from Phase I to Phase II (985) than that from Phase II to Phase III (151) (Figure 3B). This indicates that the transcriptional profile changed more dramatically from early H_2_ photoproduction to peak H_2_-photoproduction rate after anaerobic induction (from Phase I to Phase II) than the time period from peak H_2_ rate to decreased H_2_-photoproduction rate (from Phase II to Phase III). These 2,633 contigs can be further divided into 8 clusters (Figure 3A). The expression levels of more than half of the 2,633 contigs within cluster 1, 2, 3 are higher in Phase I than in Phase II and Phase III. The expression levels for the rest of the 2,633 contigs increased, with the exception of the 36 contigs in Cluster 8 whose expression levels decreased at the early H_2_-photoproduction phase and increased at late phase, with a decreased H_2_ production rate (Figure 3A).

During the time period from Phase I to Phase II, 985 contigs were significantly differentially-expressed with at least 2-fold changes, comprising 572 upregulated and 413 downregulated. Only 151 contigs were differentially expressed from Phase II to Phase III, with 64 upregulated and 87 downregulated (Figure 3B, Additional file 9A, 10). Thirty-eight contigs were significantly differentially-expressed in both comparisons of Phase I/Phase II and Phase II/Phase III (Figure 3B, Additional file 9A), 8 contigs were upregulated from Phase I to Phase II and 6 of them were also upregulated from Phase II to Phase III. The remaining 30 contigs were downregulated from Phase I to Phase II, and 21 of these were also downregulated from Phase II to Phase III (Additional file 9A). However, most of these are hypothetical proteins with unknown function. In addition, the downregulated contigs have more GO functional assignments than that of upregulated contigs. The downregulated contigs are involved in catabolic and biosynthetic metabolic process, transportation, and response to stimulus, showing protein binding, and catalytic activities within the cytoplasm, plastid, and mitochondrion cellular components (Additional file 9A). Interestingly, the expression patterns of the 26 common contigs in all three comparisons were the same, with 5 contigs upregulated and the rest downregulated. However, the expression patterns for the 12 contigs that were only common in Phase I/Phase II and Phase II/Phase III comparisons were behaved in the opposite manner. Three contigs were upregulated in the Phase I/Phase II comparison but downregulated in the Phase II/Phase III comparison, and vice versa for the rest 9 contigs (Figure 3B, Additional file 9A).

### Comparison with other green algae with reference genome sequences

Contigs generated in this study for *C*. *moewusii* were compared to the transcripts of four other green algae with reference genome sequences (*C*. *reinhardtii*, *C*. *variabilis* NC64A, *V*. *carteri*, and *C*. *subellipsoidea*) by BlastX and BlastN to identify the common transcripts these species possess, as well as their function and expression patterns during H_2_ evolution.

Compared with BlastX results, BlastN results retrieved fewer hits (Table 2). For example, 5,386 *C*. *moewusii* contigs matched 3,886 *C*. *reinhardtii* transcripts for BlastN when the cut-off E-value was set at 10^-6^ compared to 14,784 *C*. *moewusii* contigs matching 7,974 *C*. *reinhardtii* transcripts with a BlastX cut-off E-value of 10^-6^. Similar patterns were also observed for the Blast results with the other three green algae at all three cut-off E-values of 0, 10^-6^, or 10^-3^ respectively (Table 2). This result indicated that the conservation level of algae is lower at nucleotide level than that of protein level, and the BlastX results were therefore used for further comparisons.

**Table 2:** **Comparisons of homologues between *C***. ***moewusii *and other four green algae with reference genome sequences by mapping**, **BlastN and BlastX**

**Algal reference**	***Cre***	***ChlN***	***Coc***	***Vca***
Reference Transcripts #	17,114	55,307	65,067	15,285
Refernce Ave Length (bp)	3,117	1,370	1,475	1,817
**Mapping**				
Mapped Transcripts #	680	162	144	304
Mapped Ave Length (bp)	957	759	773	818
Percentage Mapped	1.99	0.47	0.42	0.89
**BlastN**				
Transcript hit (E-value = 0)	135 (126)^*^	17 (17)	15 (15)	38 (38)
Transcript hit (E-value < =10^-6^)	**5**,**386** (**3,886**)	**2**,**767** (**2**,**090**)	**2**,**184** (**1**,**712**)	**3**,**749** (**2**,**818**)
Transcript hit (E-value < =10^-3^)	14,106 (8,111)	8,559 (4,797)	4,855 (3,323)	10,869 (6,098)
**BlastX**				
Transcript hit (E-value = 0)	972 (876)	492 (466)	942 (849)	633 (591)
Transcript hit (E-value < =10^-6^)	**14**,**784** (**7**,**974**)	**10**,**948** (**7**,**355**)	**13**,**615** (**7**,**053**)	**10**,**646** (**7**,**266**)
Transcript hit (E-value < =10^-3^)	19,992 (9,735)	14,953 (8,945)	17,960 (8232)	12,956 (8181)

*The number before and within brackets mean: the number of *C*. *moewusii* transcripts with Blast hits from reference alga and the number of reference transcripts hit by *C*. *moewusii* query transcripts. ***Cre****C*. *reinhardtii*, ***ChlN****C*. *variabillis* N64A, ***Coc****C*. *subellipsoidea*, and ***Vca****V*. *carteri*.

**Figure 4:**
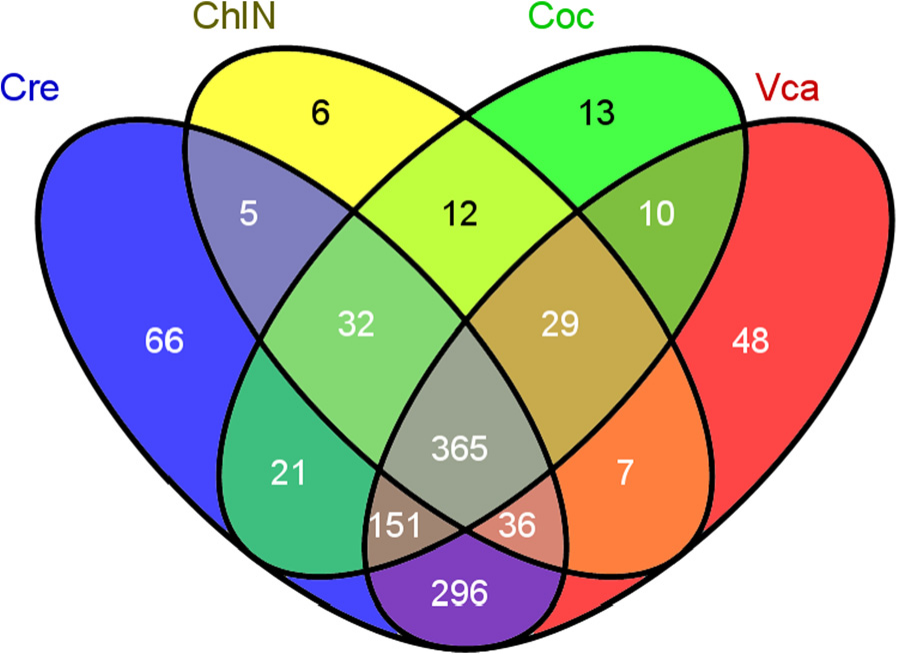
**Venn Diagram of the numbers of *C*. *moewusii *transcripts with BlastX hits from other four green algae with reference genome sequences.** The BlastX cut-off value is 0 for this comparison. ***Cre***: The number of *C*. *moewusii* transcripts with BlastX hits to *C*. *reinhardtii*; ***ChlN***: The number of *C*. *moewusii* transcripts with BlastX hits to *C*. *variabillis* N64A; ***Coc***: The number of *C*. *moewusii* transcripts with BlastX hits to *C*. *subellipsoidea*; and ***Vca***: The number of *C*. *moewusii* transcripts with BlastX hits to *V*. *carteri*.

With the decrease of E-value stringency from 0 to lower than 10^-6^ and 10^-3^, the BlastX hits number for each transcript increased and the percentage of total contigs matching reference sequences increased (Table 2). To exclude the possible false positives, only those contigs with E-value number of 0 from BlastX results were used for comparisons. The results indicated these 5 green algae have high similarity with the majority of 365 contigs held in common among all species (Figure 4, Additional file 11 A). In addition, only 54 of 492 contigs shared between *C*. *moewusii* and *C*. *variabilis* NC64A are different from those found in *C*. *reinhardtii*, with the remaining 438 contigs common to these two organisms. Similar results were found in all other pairwise comparisons (Figure 4).

Most of the 365 contigs common to all 5 strains were functionally conserved with multiple GO and enzyme code assignments, but their expression was not significantly different during the H_2_-photoproduction process. However, one transcript (contig_4525 encoding a deoxypusine synthase 1 with potential role in controlling cell proliferation and growth) was upregulated when H_2_-photoproduction decreased, and 48 other contigs (4 upregulated and 44 downregulated) were significantly differentially-expressed from the beginning of anaerobic induction to the end of the H_2_-photoproduction process (Phase I to Phase III). Nine contigs in this latter category were also significantly differentially-expressed (1 upregulated and 8 downregulated) from Phase I to Phase II and one was also downregulated from Phase II to Phase III (Additional file 11A). The other 4 upregulated contigs 3700, 2894, 2517, and 4004 were annotated as aldehyde dehydrogenase, kinesin-like calmodulin binding protein, dynein heavy chain 9, and one hypothetical stress-response protein, respectively. The expression pattern of contig_3700 (a *C*. *reinhardtii* aldehyde dehydrogenase homolog) was similar to the qPCR result observed for *C*. *reinhardtii* ADHE gene expression, with a dramatic increase in the early stage after anaerobic induction, as reported previously [10]. Dynein heavy chain 9 and kinesin-like calmodulin binding protein may be related to flagellar functions as well as cell division [46,47]. Most of the downregulated contigs are hypothetical proteins within different cellular components (e.g. cytoplasm, mitochondrion, plastid, and peroxisome) with diverse functions including binding, catalytic, hydrolase, peptidase, transferase and motor activity, and involved in various biological processes such as carbohydrate metabolism, ion transport, stress response, cellular protein modification, and translation. Four downregulated contigs are related to chaperonin complex component, and 5 related to 26S proteasome regulatory complex (Additional file 11A).

Since both *C*. *reinhardtii* and *C*. *moewusii* are hydrogen-evolving chlorophytes and *C*. *reinhardtii* is the model strain [9,27], further detailed comparison at the pathway level between *C*. *reinhardtii* and *C*. *moewusii* was carried out using both the Algal Functional Annotation Tool, a web-based comprehensive data-mining suite integrating annotation data from several pathway, ontology, and protein family databases for algal genomics [48], and ChlamyCyc, an integrative systems biology database and web-portal for *C*. *reinhardtii*[49]. Based on the statistics data from Algal Functional Annotation Tool website, *C*. *reinhardtii* has 266 KEGG pathways, which is similar to the number of pathways (258 pathways) in ChlamyCyc database [48,49]. With a cut-off E-value of 10^-3^, 19,992 *C*. *moewusii* contigs matched to 9,735 *C*. *reinhardtii* transcripts, and when the cut-off E-value stringency increased to 10^-6^, *C*. *moewusii* had 14,784 contigs matching to 7,974 *C*. *reinhardtii* transcripts (Additional file 11B). These datawere used as query ID for functional characterization mapped to 246 KEGG, 141 Panther, 438 MetaCyc, and 68 Reactome pathways respectively (Additional file 11C) covering most of the 266 KEGG pathways of the model green alga *C*. *reinhardtii*, of which purine metabolism pathway had the most hits of 176 contigs (Additional file 12A) followed by pathways for plant hormone biosynthesis, and spliceosome-related genes (Additional file 11C, 12B). Other KEGG pathways with multiple transcript hits encoding for a nearly complete pathway or complex include pyrimidine metabolism, biosynthesis of alkaloids, terpenoids and steroids, carbon fixation, glycolysis/gluconeogenesis, pyruvate metabolism, TCA cycle and oxidative phosphorylation, fatty acid biosynthesis, RNA polymerase, ribosome, and protein export system (Additional file 11C, 12C). These results indicate that *C*. *moewusii* and *C*. *reinhardtii* share multiple core functional pathways.

In addition, if combined with the protein abundance information, mRNA abundance can help us understand the post-transcriptional regulation [50,51] and feature of evolutionary conserved proteins which will be briefly discussed below for transcripts shared among five green algae. The average of all the RPKM in three different phases of Phase I, II, and III for each transcript was calculated and used to represent the transcript expression abundance, which ranged from a minimum of −3.68 to a maximum value of 15.19 with a mean of 5.01 (Additional file 5C). Based on statistical analysis of RPKM abundance for the 34,136 contigs, 34,124 had expression value and 856 contigs had the top 2.5% RPKM value accounting for almost 9% of the total expression abundance from all 34,124 contigs (Additional file 11D). A similar number of contigs (875) had the lowest RPKM values, among which 236 contigs are homologous to 180 *C*. *reinhardtii* proteins based on BlastX cut-off E-value of 10^-6^ (Additional file 11D). However, only a small percentage of these contigs was significantly differentially-expressed in at least one comparison among Phase I, II, and III, which included 38 contigs with highest abundance and 43 contigs with lowest abundance (Additional file 11D).

In contrast to the contigs with lowest abundance, the most abundant top 2.5% contigs had a higher number of homologues to *C*. *reinhardtii*, with 612 *C*. *moewusii* contigs matching 512 *C*. *reinhardtii* proteins at the same cut-off E-value of 10^-6^ (Additional file 11D). Correspondingly, the most abundant contigs were tracked to more metabolic pathways than contigs with the lowest abundance (Additional file 11E). Similarly, the contigs with the most abundant reads had more positive hits to the KEGG pathways database, GO term, and MapMan ontology numbers than the scarcer ones. In addition, the number of contigs within a particular pathway was also higher for the abundant contigs than for the scarcer ones, and this is not related to enzyme number within the pathways. For example, 78 high-abundance contigs had hits in the ribosome complex and 27 had hits in the pathways of biosynthesis of phenylpropanoids and biosynthesis of plant hormones. Conversely, the top 2 pathways for low-abundance contigs only contained 5 and 4 hits to purine metabolism and nitrogen metabolism pathways, respectively, which suggest the conserved feature of the abundant contigs and the importance of these contigs in anaerobic metabolism (Additional file 11E).

### Expression pattern of hydrogenase and hydrogenase assembly genes

To identify *C*. *moewusii* hydrogenase contigs related to H_2_ evolution, protein sequences of HYDA1 (Genbank accession: AAT90438.1) of *C*. *moewusii* and four hydrogenase proteins of *C*. *reinhardtii* (HYDEF: XP_001691465.1; HYDG: XP_001691319.1; HYDA1: XP_001693376.1; HYDA2: XP_001694503.1) were Blasted against the 34, 136 *C*. *moewusii* contigs using tBlastN, and the 34, 136 *C*. *moewusii* contigs were also Blasted against these 5 protein sequences mentioned above using BlastP. The Blast results identified *C*. *moewusii* contigs homologous to the two *C*. *reinhardtii* hydrogenases, HYDA1 and HYDA2, as well as to the two hydrogenase assembly proteins, HYDEF and HYDG (Additional file 9B). In addition, 88,159 sequences with annotation related to hydrogenase were downloaded from NCBI database and used as query sequences to Blast against the 34,136 *C*. *moewusii* contigs. Besides the 4 hydrogenase-related genes identified above, 2 other contigs (contig_33705 and contig_27051) were homologous to the fragment of *C*. *reinhardtii* HYD3 (XP_001693369, 479-aa) with short length of 501-bp and 331-bp respectively. However, HYD3 is a NarF-like protein involved in Fe-S cluster assembly rather than H_2_ production [52,53]. We therefore focus on the 4 conserved hydrogenases and hydrogenase assembly proteins.

*C*. *moewusii* contig_2138 and contig_839 have high similarity to the *C*. *reinhardtii* hydrogenase-assembly proteins HYDEF and HYDG, respectively, and were therefore assigned as HYDEF and HYDG for *C*. *moewusii* (Additional file 9B). The amino acid sequence based on contig_745 is homologous to that of *C*. *reinhardtii* hydrogenase HYDA1 [54], and it also has strong homology with the *C*. *reinhardtii* hydrogenase HYDA2 [55] with a better E-value of 9E-177 than that of contig_1013 (3E-166). Since contig_745 matches the published *C*. *moewusii* hydrogenase HYDA1 perfectly, with the exception of two amino acid changes (V53A and A424V) and the lack of the last amino acid E at the C-terminal, it was assigned as HYDA1 and contig_1013 as HYDA2. Based on the assembled contig information of these 2 contigs, the 5′ UTRs of HYDA1 and HYDA2 are 105 and 126-bp, similar to those for *C*. *reinhardtii* hydrogenases [56], and slightly longer than the 74-bp of 5′ UTR as reported before for HYDA2 by Rapid Amplification of cDNA Ends (RACE)-PCR [57]. Recently, a novel hydrogenase has been identified and characterized in *Chlorella variabillis* NC64A with an ancestral form of accessory F-cluster (FeS cluster binding domains) besides the classical algal hydrogenase H-cluster (HYDA active site) [58]. Similar to the hydrogenases of *C*. *reinhardtii*, the *C*. *moewusii* hydrogenases HYDA1 and HYDA2 identified in this work contain the H-cluster only, which is not surprising since *C*. *reinhardtii* and *C*. *moewusii* are from same *Chlamydomonadaceae* family with close phylogenetic relationship. It is also consistent with the previous phylogenetic analysis result of the loss of HYDA F-clusters in chlorophycean algae [58].

The expression of 2 contigs (contig_745 and contig_1013) annotated as [FeFe]-hydrogenase *HYDA1* and *HYDA2*[57] matched the pattern of H_2_ evolution, increasing during early H_2_ production and decreasing when the H_2−_production rate decreased (Additional file 9B). This is similar to a previous report of hydrogenase (HYDA) gene expression following anaerobic induction in *C*. *reinhardtii*[54-56,59]. Notably, *HYDA1* (contig_745) had an elevated expression level of log_2_-based RPKM value after 15 min induction jumping from 1.41 at time 0 to about 9, which is more than a 200-fold increase. The expression of the other hydrogenase and the 2 hydrogenase assembly genes also increased at least 3-fold from time 0 to 15 min post-induction (Phase I). After 15-min post-induction, the changes were mostly associated with the two hydrogenases (contig_745 and contig_1013), with at least a 2-fold increase from Phase I to Phase II and a 3-fold decrease from Phase II to Phase III (Additional file 9B). However, due to the limited sample size and stringent statistical standard, the changes observed from Phase I to Phase III are not statistically significant, and further investigation into the transient change immediately after induction is needed. Combining with the fact that these 2 contigs were also highly expressed with large RPKM values (Additional file 9B), we propose that these are the hydrogenases responsible for H_2_ production in this organism and that HYDA1 is the major hydrogenase inducible by the anoxia as the electron acceptor for reducing equivalents generated during fermentation. All [FeFe] hydrogenases studied to date are oxygen sensitive and irreversibly inactivated by trace amounts of oxygen [56,58,60-62]. The relatively abundant expression level of *HYDA2* (contig_1013) even at time 0 before the anaerobic induction suggests that the two hydrogenase genes are differentially regulated, and further investigations of the factors and conditions responsible for transcription of *HYDA1* and *HYDA2* genes during anaerobic induction could help us understand the synergetic function between these two hydrogenase paralogues and their roles on H_2_-photoproduction (Additional file 9B).

### Transcriptional differences between *C*. *moewusii* and *C*. *reinhardtii* in the fermentative pathway

Besides measuring hydrogenase activity, Meuser et al. (2009) also compared starch catabolism and secreted metabolites of *C*. *moewusii* strains with those of *C*. *reinhardtii* during dark anaerobic fermentation [9]. The results indicated that the *C*. *moewusii* SAG 24.91 strain had faster rates of dark anaerobic starch catabolism than *C*. *reinhardtii*, with different profiles of fermentation end-products. Formate, acetate and ethanol are the dominant anaerobic metabolites of *C*. *reinhardtii* CC-124 strain with a ratio of 2:1:1 or 2:2:1 depending on the strain, culture and assay conditions [9,10]. Instead, the primary fermentation products of *C*. *moewusii* SAG 24.91 are acetate, ethanol and glycerol, with an approximate ratio of 2:1:1 after 4 h of anaerobiosis [9].

**Figure 5:**
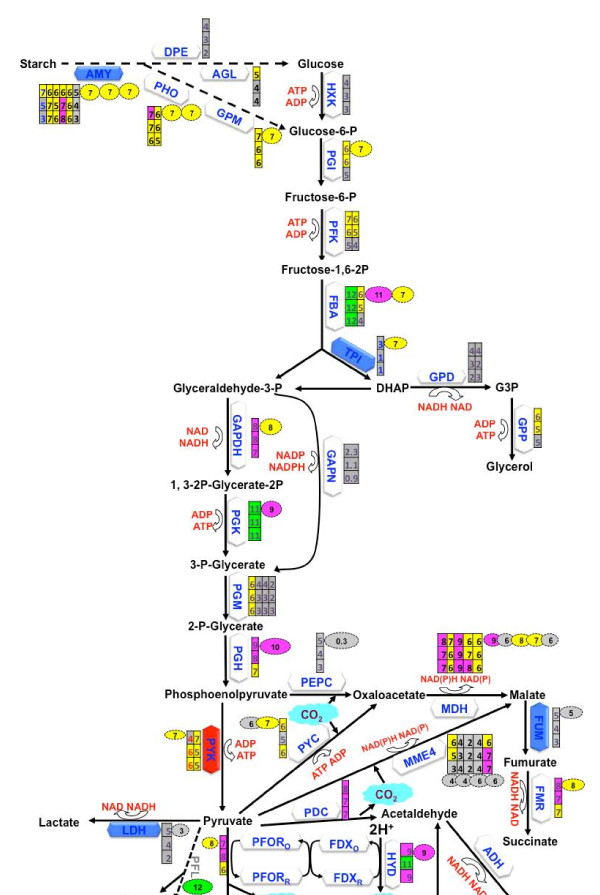
**Starch catabolism**, **glycolysis and anaerobic fermentation pathways in *C. moewusii *constructed based on the information from *C. reinhardtii *and the transcript abundance of enzymes in *C. moewusii *and *C. reinhardtii.*** Enzymes are represented by gray diamond boxes, except for PFL, whose gene sequence has not been identified in *C*. *moewusii*. The blue diamond boxes indicate that the respective gene is downregulated, and red color indicates upregulation. The numbers within the stacked rectangle boxes indicate the abundance of the respective transcript at Phase I, II and III, from top to bottom, respectively. Multiple columns were included for enzyme with multiple homologues. The numbers within the oval boxes represent the transcriptional abundance of homologous transcripts in *C*. *reinhardtii*. The abundance of each transcript is also represent by the color of the filled boxes, with green, pink or yellow indicating, respectively, the top 0.5%, 2.5% or 10% of all 1 transcripts, respectively.

To connect the transcriptomic data from this RNA-Seq study with previous *C*. *moewusii* metabolite profile, the starch catabolism, glycolysis and pyruvate fermentation pathways were reconstructed (Figure 5) based on previous reports [9,10,23,58,63-67] and ChlamyCyc database [49]. The model species *C*. *reinhardtii* is well studied, with 261 Sequence Reads Achieve (SRA) datasets (totally 1150 Gb) available at NCBI. However, we searched the SRA database and only identified one experiment actually contains RNA-Seq result using samples from 45 min and 120 min post anaerobic induction in the dark for the same *C*. *reinhardtii* strain (CC-124) used in our reference study [9]. The dataset (SRR057469) for this sample (SRS074576) was downloaded and RNA-Seq analysis result for 15, 935 *C*. *reinhardtii* reference mRNA was generated. The transcriptional abundances based on log_2_-transformed RPKM values during anaerobic fermentation for *C*. *moewusii* and *C*. *reinhardtii* were compared to understand the metabolite profile difference at the transcriptional level.

We couldn’t identify any significant pyruvate-formate lyase (PFL) homologue of *C*. *reinhardtii* in the 34,136 contigs generated in this study of *C*. *moewusii* (Additional file 13A), which agrees with the previous hypothesis that the absence of formate in the secreted metabolites of *C*. *moewusii* is due to the lack of PFL enzyme activity [9]. In addition, the PFL transcript of *C*. *reinhardtii* is upregulated after anaerobic dark induction [10], with a log_2_-transformed RPKM value of 11.5 (top 0.5% among all the 15,935 *C*. *reinhardtii* reference mRNA), which may account for the dominance of formate in the secreted metabolites in *C*. *reinhardtii* as previously reported [9]. Other enzymes with different abundance levels between *C*. *moewusii* and *C*. *reinhardtii* are glycerol-3- phosphatase (GPP), phosphate acetyltransferase (PAT) and acetate kinase (ACK), which may account for the relative higher product ratios of acetate and glycerol in *C*. *moewusii* compared to *C*. *reinhardtii* (Figure 5, Additional file 13A).

Except for the absence of PFL gene, *C*. *moewusii* possesses similar metabolic flexibility as it produces glycerol, lactate, succinate, ethanol, and acetate. This flexibility may help *C*. *moewusii* adapt to different environmental conditions, as suggested for *C*. *reinhardtii* (Figure 5). In contrast to the upregulation of several *C*. *reinhardtii* genes encoding PFOR, PTA2, ACK1 and amylase after anaerobic induction [10], only 5 contigs among all these enzymes involved in starch catabolism, glycolysis, and anaerobic fermentation in *C*. *moewusii* are significantly differentially-expressed from Phase I to Phase III at the transcriptional level. The only upregulated transcript is contig_683, encoding a pyruvate kinase (PYK). Other 4 contigs encoding an amylase (contig_9910), triose phosphate isomerase (TPI, contig_9498), phosphoenolpyruvate carboxylase (PEPC, contig_5043), and D-lactate dehydrogenase (LDH, contig_14499) were downregulated significantly (Figure 5, Additional file 13A). Considering the different amylase gene expression patterns and starch utilization rate between *C*. *reinhardtii* and *C*. *moewusii*[9,10], it will be interesting to investigate the correlation between the amylase gene expression and starch utilization rate. Notably, three of the downregulated genes are directly related to enzymatic NADH-requiring reactions which may be responsible for redox balancing under anaerobic conditions (Figure 5). The downregulation of these genes may therefore redirect the electron reductant from NADH to the hydrogenase reaction for increased H_2_ photoproduction (Figure 5), and they could be potential genetic candidates for metabolic engineering to increase H_2_ photoproduction.

However, the implementation of metabolic pathway engineering towards blocking NADH-utilizing pathways as a means of improving fermentative H_2_ production has not yielded desired results so far [63-65]. For example, although the *adh1 C*. *reinhardtii* mutant, which lacks the alcohol dehydrogenase gene is unable to produce ethanol, it redirects its metabolism towards other alternative NADH-utilizing pathways such as those involved in glycerol and lactate production, instead of towards H_2_ production, which is unchanged in this mutant [63]. Similarly, the disruption of formate production [64,65] as well as double disruption of formate and ethanol production [64] in *C*. *reinhardtii* also results in rerouted fermentative pathways, yielding changed metabolite profiles, especially for those metabolites that require NADH as reductant. These mutants show increased levels of lactate, ethanol or glycerol. However, dark fermentative H_2_ production decreased in both the *pfl1*-*1* single mutant and the double mutant (*pfl1*-*1 adh1*) [64,65]. The results indicate the close connection between fermentation and H_2_ production. Therefore it is very important to consider electron transport pathways [68] and redox balancing for the H_2_-photoproduction improvement through metabolic engineering.

The lack of PFL and accompanying accumulation of glycerol in *C*. *moewusii*, as well as the accumulation of glycerol and lactate in both the *adh1* and the *pfl1*-*1 adh1 C*. *reinhardtii* mutants also indicate that glycerol and lactate production could be the major routes for redox re-balancing in green algae. It will be interesting to investigate the role of glycerol in other algae besides the well-studied species such as *C*. *reinhardtii*, since the potential for glycerol production is believed to be widespread among algae [66]. The existence of alternative fermentative pathways could help green algae to adapt to different environments, and the downregulation of genes involving in metabolic pathways for glycerol, succinate, and lactate production during H_2_ photoproduction may be one of the reasons for the high H_2_ photoproduction in *C*. *moewusii*, besides the rapid increase in *HYDA1* gene expression discussed above from time 0 to Phase I. In addition, the differences in hydrogenase expression between *C*. *reinhardtii* and *C*. *moewusii* may help explain the observed discrepancies in H_2_ photoproduction: the expression of *HYDA1* and *HYDA2* in *C*. *reinhardtii* increased, respectively, ca 20% and 50-fold in response to anoxia [69], while the expression of *HYDA1* in *C*. *moewusii* increased more than 200-fold and that of *HYDA2* only 4.5-fold from time 0 to Phase I (15 min post-induction).

Although enzymes involved in fermentation and anaerobic respiration and their expression have been intensively studied, especially within the model species (e. g. *C*. *reinhardtii*), only a few reports on regulators controlling the expression of hydrogenases and fermentative pathway genes despite of growing interests [25,70-72]. The Copper Response Regulator 1 (CRR1), a SQUAMOSA promoter binding protein (SBP) family carrying a characteristic zinc finger domain that interacts with the cis-acting DNA sequence GTAC, has been demonstrated to be involved in the transcriptional activation of *C*. *reinhardtii* hydrogenase gene *HYDA1* and the ferredoxin-encoding gene *FDX5* in the absence of copper (Cu) or oxygen [70,71]. A *C*. *reinhardtii* CRR1 homologue (contig_4501) was identified in *C*. *moewusii* with high similarity (Evalue = 0), and, although the differential expression of CRR1 homologue contig_4501 was not significant, the expression level of RPKM values decreased from Phase I to Phase III (Additional file 13A). CRR1 is homologous to carbon catabolite repressor protein 4 (CCR4), which forms a conserved 9-subunit CCR4-NOT complex throughout the eukaryotic kingdom with broad roles in gene regulation [73]. For example, the yeast CCR4 protein is required for the alcohol dehydrogenase gene *ADH2* expression, and ADH2 expression was shown to be decreased in *CCR4* mutant [74]. It is possible that persistent anoxia reduced the expression of this CRR1 homolog leading to decreased ethanol production while re-routing more reducing equivalent to faster H_2_ photoproduction in *C*. *moewusii*.

In addition, metabolic responses of *C*. *reinhardtii* to sulfur-deprivation-induced anoxia have also been well studied [13,25,75-81]. Most of the sulfur-deprivation responses are controlled by several transcriptional regulatory Ser/Thr kinases belonging to the protein family of SNF1 Related Protein Kinase (SNRK) such as SNRK2.1, SAC1 and SAC3 [25,79,80]. A model for sulfur deprivation–responsive gene regulation among the three regulatory proteins SAC1, SNRK2.1 and SAC3 (same as SNRK2.2) has been proposed: SNRK2.2 inhibits SNRK2.1-activated expression of sulfur-responsive genes, and the active SAC1 unblocks the inhibition of SNRK2.1 by SNRK2.2 in sulfur-deficient conditions for full expression of sulfur-responsive genes [25,81]. SNRK2.1 and SNRK2.2 are plant-specific SNF1-like Ser/Thr protein kinases, which are conserved in eukaryotes, and the essential role of yeast Snf1 on transcriptional control of glucose-repressed genes is well characterized [82-86]. SNRK2.1 also regulated the H_2_ production-related genes, with the expression level of *HYD1*, *HYDEF*, *PFR1* and *FDX5* upregulated in the *snrk*2.1 mutant compared to its parental strain [25].

Fifty proteins are classified as SNRKs in *C*. *reinhardtii*, based on iTAK database 13.03 [87]. BlastX identified 28 contigs of *C*. *moewusii* that are homologous to these 50 SNRKs, and the expression values of 15 of them with complete Serine/Threonine protein kinase catalytic domain were extracted (Additional file 13B). Three of these contigs are upregulated and 7 downregulated at levels greater than 2-fold. Contigs 21466 and 8046 were significantly downregulated during the transition from Phase I to Phase II (contig_8046 was also downregulated from Phase I to Phase III), and contig_2540 was the only one that was significantly upregulated from Phase I to Phase III. Contig _11669, which is homologous to the *C. reinhardtii* SAC3 (SNRK2.2), was downregulated from Phase I to Phase II and Phase I to Phase III but not significantly (Additional file 13A). The existence of multiple SNRKs with different expression patterns indicates the importance of SNRKs on transcriptional control in *C. moewusii,* although further work especially genetic studies are needed to fully understand the roles of SNRKs on H_2_ photoproduction and anaerobic fermentation in *C. moewusii.*

Finally, enzymes related to starch catabolism, glycolysis, and anaerobic fermentation, as well as transcriptional regulators related to anaerobic fermentation were successfully identified for *C*. *moewusii* with high similarity to those in *C*. *reinhardtii*, with the exception of the absence of PFL and the low similarity of the pyvurate dehydrogenase (PDC) homologue (E-value of 1.58E-7) to that found in *C*. *reinhardtii*. In addition, several enzymes in *C*. *moewusii* contain multiple homologues (e.g., fructose-1,6-biphosphate aldolase (FBA), pyruvate kinase (PYK), MDH, MME4, and amylase) to those of *C*. *reinhardtii*, and the transcriptional levels for most of the enzymes involved in this pathway are very high, such that nearly all of them are among the top 10% contigs, with several among the top 2.5% and even top 0.5% (Figure 5, Additional file 5C, 13B). These results suggest that (i) *C*. *moewusii* and *C*. *reinhardtii* have inherited similar anaerobic fermentation and H_2_-photoproduction pathways from their ancestor, which underscores the importance of these pathways in green algae; (ii) the existence of different enzymes and the differential regulation at the transcriptional level between *C*. *moewusii* and *C*. *reinhardtii* may contribute to their metabolic difference; (iii) the enzymes that had the most abundant transcriptional levels in both *C*. *reinhardtii* and *C*. *moewusii* (e.g. FBA, phosphoglycerate kinase (PGK), phosphoglycerate dehydratase (PGH), and HYD) may be responsible for key metabolic steps (Figure 5, Additional file 13A).

## Conclusions

RNA-Seq data of time course transcriptomics for an unsequenced green alga *C*. *moewusii* were analyzed, providing not only a transcriptomic RNA-Seq dataset, but also biological insights on the expression patterns of contigs associated with starch catabolism, glycolysis, anaerobic fermentation, and H_2_ photoproduction during dark anaerobic induction. *C*. *moewusii* and *C*. *reinhardtii* share core functional pathways for H_2_ photoproduction and anaerobic fermentation with metabolic flexibility. The difference between *C*. *moewusii* and *C*. *reinhardtii* on hydrogenase expression and anaerobic fermentative pathways involved in redox balancing may contribute to the different profiles of hydrogenase activity and secreted anaerobic metabolites observed in each species. Even at this cursory level of analysis, it is clear that the application of this approach can lead to the generation of interesting hypotheses for both fundamental and applied research. In addition, the identification of common transcripts among different green algae and their differential expression during H_2_ production may help elucidate the physiology and evolution of thechlorophyceans.

## Materials and methods

### Strain and growth conditions

*C*. *moewusii* SAG 24.91 was kindly provided by Dr. Matthew Posewitz at the Colorado School of Mines. Tris-Acetate-Phosphate (TAP) plate and liquid medium was used for culturing the cells. To generate cultures for H_2_ measurement and RNA harvesting, *C*. *moewusii* SAG 24.91 cultures were grown in 1-L Roux bottles using TAP media at 25°C, and illuminated continuously with white fluorescent light (200 μmol photons m^-2^ s^-1^). Cultures were supplemented with 2% CO_2_ in air and continuously mixed with a stir bar. Cells were harvested for dark anaerobic induction at chlorophyll concentrations of 14 – 23 μgmL^-1^. Chlorophyll concentration was determined spectrophotometrically following extraction in 95% ethanol [9].

### Dark anaerobic induction

Liquid cultures grown in Roux bottles were centrifuged at 3000 × *g* for 1 min and resuspended in 0.1 volume of AIB buffer (50 mM potassium phosphate, pH 7.2, 3 mM MgCl_2_). Resuspended cells were transferred to 13-mL glass serum vials and sealed with butyl rubber septa. Vials were wrapped in aluminum foil and purged with argon to establish dark anaerobiosis. Cells were sampled at various time points prior to and following initiation of anaerobic conditions (Figure 1).

### H_2_ evolution measurement

A Clark-type electrode system (ALGI LLC, CO) was used to measure rates of H_2_ production using calibrated electrodes (YSI Incorporated, OH) in a water-jacketed glass chamber (25°C) illuminated with 2000 μmol photos m^-2^ s^-1^. Anaerobically induced cell suspensions were sampled approximately at 0, 10, 110, 300, 600 min, using a gas-tight syringe (Hamilton Company, NV) purged with argon. For each measurement, the chamber was filled with 1.8 mL of MOPS buffer (50 mM, pH 6.8), and then purged with argon and monitored for O_2_ using a calibrated electrode. Cell suspension (0.2 mL) was added to chamber, and H_2_ production rates were assessed from the steepest slope following illumination.

### Total RNA extraction and RNA-Seq

RNA samples were extracted approximately 5 min following H_2_ measurement using a gas-tight syringe. Samples were immediately centrifuged and RNA was extracted with Plant RNA Reagent, following the manufacturer’s instructions (Life Technologies, NY). RNA was further purified using a Turbo DNA-free kit (Life Technologies, NY) and a Qiagen RNeasy Plant Mini Kit (Qiagen, CA), followed by checking sample quality and quantity with gel electrophoresis and NanoDrop spectrophotometer (Thermo Scientific, DE). Samples of total RNA were sent to the National Center for Genomic Resources (NCGR) for RNA-Seq using Illumina GA IIx platform according to the manufacturer’s instructions. Briefly, mRNA was selected using oligo(dT) probes and then fragmented. cDNA was synthesized using random primers, modified and enriched for attachment to the Illumina flowcell. The fluorescent images process to sequences, base calling and quality value calculation were performed by the Illumina data processing pipeline (version 1.5).

### Reference contigs determination and their functional annotation

Seven raw fastq datasets generated through Illumina GA IIx were imported into FastQC software (http://www.bioinformatics.babraham.ac.uk/projects/fastqc/) for quality assessment. The X-axis on the FasrQC output graph of Per Base Sequence Quality shows the read position (bp), and Y-axis shows the quality scores with higher scores corresponding to better base calls. The Y-axis was divided into three regions with green background for very good quality calls (Quality score > =28), orange background for calls of reasonable quality (20 < =Quality score < 28), and red background for poor quality base calls (Quality score < 20). The raw fastq datasets were then trimmed using CLC Genomics Workbench (version 5.1, CLCBio, Denmark) before de novo assembly was carried out using CLC Genomics Workbench. The parameter used for data trimming are: Quality trim limit = 0.05; Ambiguous trim limit = 2; Minimum number of nucleotides in reads = 15; Discard short reads = Yes; Discard long reads = No; Remove 5′ terminal nucleotides = No; Number of 3′ terminal nucleotides Removed = 9. In addition, the duplicate reads within the trimmed data were also removed using CLC Genomics Workbench and the seven trimmed data without duplicate reads as well as seven original raw data without data trimming and duplicate removal were used as input data for de novo assembly. The parameters used for de novo assembly are: Mapping mode = Map reads back to contigs (slow); Similarity fraction = 0.8; Minimum contig length = 200; Mismatch cost = 2; Insertion cost = 3; Deletion cost = 3; Update contigs = Yes; Length fraction = 0.5; Perform scaffolding = Yes.

Blast2GO [40,41] software was used for BLASTX, GO term and enzyme code annotation, InterProScans and KEGG pathway analysis of the contigs. InterProScans was performed against all of the available InterPro databases, and KEGG pathway information was retrieved based on the enzyme commission (EC) numbers [88-90]. Blast2GO annotation results were used to generate the combined graphs of biological process, molecular function, and cellular component. In addition, BLASTX to a non-redundant (nr) protein database from the NCBI GenBank database [91,92] was used to assign the top homologous hits to the assembled contigs. Finally, to compare the difference among *C*. *moewusii* and other microalgae with sequenced genomes including *C*. *reinhardtii*, *V*. *carteri*, *C*. *variabilis*, and *C*. *subellipsoidea*, *C*. *moewusii* contigs were used as query sequence for BLASTN and BLASTX search against the transcript and protein sequences of these microalgae respectively. The cutoff E-value threshold is set arbitrarily to < = 10^-6^.

### RNA-Seq data processing and statistical analysis

Six trimmed RNA-Seq datasets were used as input sequencing reads and the 34,136 contigs generated through de novo assembly using trimmed data with duplicates removed were selected as the reference. RNA-Seq analysis was carried out using CLC Genomics Workbench with the settings described below: Minimum read count fusion gene table = 5; Minimum length of putative exons = 50; Minimum number of reads = 10: Maximum number of mismatches (short reads) = 2; Unspecific match limit = 10; Additional upstream bases = 0; Minimum exon coverage fraction = 0.2; Minimum length fraction (long reads) = 0.9; and Minimum similarity fraction (long reads) = 0.8.

Eexpression levels of RPKM values for each transcript from each RNA-Seq dataset were exported and log_2_ transformed, which were then combined to provide the RNA-Seq data for statistical analysis using JMP Genomics 6.0 (SAS Inc., NC) as described before for data quality control, data normalization, One-Way ANOVA analysis, and K-means Clustering [43-45]. The information of assembled contigs of Transcriptome Shotgun Assembly (TSA), Sequence Reads Archive (SRA), and Gene Expression Omnibus (GEO) has been submitted to NCBI for this study with the accession of SRP021194 and GSE46225 for SRA and GEO respectively.

### Comparisons among other green algae with reference genome sequences

The FASTA files of transcripts and proteins of sequenced *C*. *reinhardtii*, *C*. *variabilis* NC64A, *V*. *carteri*, and *C*. *subellipsoidea* were downloaded from JGI website (http://www.jgi.doe.gov) and imported into CLC Genomics Workbench. These were used as reference sequences for Local Blast using the 34,136 *C*. *moewusii* transcripts as query sequences. Two Blast programs were used, BlastX using predicted protein sequences as reference and BlastN using transcripts as reference sequences. *C*. *moewusii* contigs with hits to each reference dataset were reported and the corresponding homologues with multiple hits were consolidated.

## Abbreviations

AMY: Amylase; DPE: Alpha-glucanotransferase; AGL: Alpha glucosidase; PHO: Starch phosphorylase; GPM: Phosphoglucomutase; HXK: Hexokinase; PGI: Phosphoglucose isomerase; PFK: Phosphofructokinase; FBA: Fructose-1,6-biphosphate aldolase; TPI: Triose phosphate isomerase; GPD: Glycerol-3-phosphate dehydrogenase; GPP: Glycerol-3- phosphatase; GAPDH: Glyceraldehyde 3- phosphate dehydrogenase; PGK: Phosphoglycerate kinase; GAPN: Glyceraldehyde-3-phosphate dehydrogenase; PGM: Phosphoglycerate mutase; PGH: Phosphoglycerate dehydratase; PYK: Pyruvate kinase; LDH: Lactate dehydrogenase; PDC: Pyvurate dehydrogenase; HYD: Hydrogenase; ADH: Alcohol dehydrogenase; PFOR: Pyruvate-ferredoxin oxidoreductase; FDX: Ferredoxin; PFL: Pyvurate formate lyase; ACS: Acetyl-CoA synthetase; PAT: Phosphate acetyltransferase; ACK: Acetate kinase; DHAP: Dihydroxy-acetone phosphate; G3P: Glycerol-3-phosphate; FMR: Fumarate reductase; FUM: Fumarase; MDH: Malate dehydrogenase; MME4: Malic enzyme; PEPC: Phosphoenolpyruvate carboxylase; PYC: Pyruvate carboxylase.

## Competing interests

The authors declare that they have no competing interests.

## Authors’ contributions

MTG, SS, MG and PTP designed the experiment. SS carried out the culturing, H_2_ measurement and RNA extraction. SY analyzed the data. SY, MTG, SS, MG, and PTP wrote the manuscript. All authors read and approved the final manuscript.

## Supplementary Material

Additional file 1 RNA-Seq data analysis 1 flowchart used in this study. Click here for file

Additional file 2 **General Information about the RNA-Seq Data.** The dataset for 15 M-0502 was originally trimmed by sequencing facility and the data with red font were therefore not included for statistical calculation of the average (AVE) and standard deviation (SD). Click here for file

Additional file 3 **An example of improved RNA-Seq reads quality after data trimming.** The ×6 axis on the FasrQC output graph of Per Base Sequence Quality shows the read position (bp), and Y-axis shows the quality scores with higher scores corresponding to better base calls. The Y-axis was divided into three regions with green background for very good quality calls (Quality score > =28), orange background for calls of reasonable quality (20 < =Quality score < 28), and red background for poor quality base calls (Quality score < 20). Click here for file

Additional file 4 **The cumulative contig length with the increase of contig numbers (A) and statistical result of the length of assembled contigs using trimmed raw data with duplicate reads removed (B).** X-axis is the number of contigs and Y-axis is the cumulative length of the contigs (kb) from contig_1 to contig_34136 (A). N: number of contigs used for statistical analysis. Click here for file

Additional file 5 **Annotation result of the 34,136 *C*. *moewusii *contigs based on BlastX and Blast2GO results (A); Top hits of all 34,136 assembled *C*. *moewusii *contigs to 40 published *C*. *moewusii *Sequences (B); Quartiles of expression value for each individual time point, phase, and all 7 datasets, as well as statistical result of time-course differential expression for all 34,136 *C*. *moewusii *contigs post anaerobic induction (C); and Blast2GO results of the functional roles of the 34,136 *C*. *moewusii *contigs in Biological Process, Molecular Function, and Cellular Component (D).** Click here for file

Additional file 6 **The relationship between *C*. *moewusii *transcripts generated through this study and the published *C*. *moewusii *mitochondrial genome sequence (NC_001872) (A), and BlastX results of two assembled contigs of contig_490 (B) and contig_1136 (C).** 34,136 contigs were mapped against NC_001872 to investigate their relationship. Contigs with green color have same orientation as the reference and contigs with red color have reverse orientation as the reference (A). The graphic of B and C is an overview of NCBI NR database sequences aligned to the query sequence of contig_490 (the longest one, 15888-bp) and contig_1136 respectively. Alignments are color-coded by score, within one of five score ranges, the larger the number, the more conserved. Multiple alignments on the same database sequence are connected by a dashed line. Click here for file

Additional file 7 **Blast2GO results of combined graphs for the distributions of 34, 136 *C*. *moewusii *transcripts involved in different levels of Biological Processes (A), Molecular Function (B), and Cellular Components (C).** And examples of enzymes identified by Blast2GO involved in pathways of One Carbon Pool by Folate (map00670) (D), C4-dicarboxylic Acid Cycle of Carbon Fixation in Photosynthetic Organism (map00710) (E). The enzymes in the pathways of D and E with colors highlighted are enzymes identified in *C*. *moewusii*, the ones without color highlighted are enzymes with no homologues identified in *C*. *moewusii*. Click here for file

Additional file 8 **Parallel plot of RNA-Seq data distribution 1 before normalization (A) and Heat map and dendrogram (B), as well as parallel plot of RNA-Seq data distribution after normalization (C), and correlation scatterplots of the biological replicates at different phases (D4 F).** (D: Phase I; E: Phase II; F: Phase III) using Pairwise method in JMP Genomics. The X-axis and Y-axis for A and C are the log2-based RPKM value and its corresponding density. Click here for file

Additional file 9 **RNA-Seq One-Way ANOVA statistical analysis result of the significantly differentially expressed 2,633 *C*. *moewusii *contigs post-anaerobic induction (A); and the differential expression of 4 contigs identified as hydrogenases and hydrogenase assembly proteins as well as the alignment of *C*. *moewusii *hydrogenases (Cmo_745_hydA1 and Cmo_1013_HydA2) and *C*. *reinhardtii *hydrogenases (Cre_HydA1 and Cre_HydA2).** Click here for file

Additional file 10 **Volcano plots of One-Way ANOVA analysis results (A: Phase I/Phase II; B: Phase II/Phase III; C: Phase I/Phase III) using JMP Genomics 6.0.** The X-axis is the log2-based ratio and Y-axis is the value of –log10P-value. The horizontal dashed red line indicates the significance level with the values above the level are statistically significant. Click here for file

Additional file 11 **RNA-Seq One-Way ANOVA statistical analysis result of the 365 *C*. *moewusii *contigs conserved among five green algae (A); BlastX result of the 34, 136 *C*. *moewusii *contigs to *C*. *reinhardtii *proteins (B); the number of *C*. *moewusii *contigs identified in different pathways (C); RNA-Seq One-Way ANOVA statistical analysis result of the 686 *C*. *moewusii *contigs with the highest log2-based RPKM mean value and 875 *C*. *moewusii *contigs with the lowest mean value and their homologues in *C*. *reinhardtii *(D); and *C*. *reinhardtii *genes identified in different pathways that are homologous to *C*. *moewusii *contigs with the highest 2.5% and lowest 2.5% of the log2-based RPKM mean value (E).** Click here for file

Additional file 12 **Examples of KEGG metabolic pathways that the 34,136 *C*. *moewusii *transcripts with hits to *C*. *reinhardtii*.** 176 *C*. *moewusii* transcripts homologous to *C*. *reinhardtii* involved in Purine Metabolism (A), 125 *C*. *moewusii* transcripts homologous to *C*. *reinhardtii* involved in spliceosome (B), and 125 *C*. *moewusii* transcripts with homologous to *C*. *reinhardtii* involved in KEGG pathway of Carbon Fixation in Photosynthetic Organisms (C). The enzyme codes with red color indicate that homologues existing in *C*. *moewusii*. The cut-off E-value is 10-6 to identify *C*. *moewusii* homologues in *C*. *reinhardtii*. Click here for file

Additional file 13 **The statistical analysis result of the distribution of 7,940 genes of *C*. *reinhardtii *with log2-based RPKM values as well as all log2-based RPKM values based on published NCBI SRA dataset SRR057469 using the *C*. *reinhardtii *RNA extracted at 45 min and 120 min post dark anaerobic induction (SRS074576) for all the 15, 935 *C*. *reinhardtii *reference mRNA (A); and RNA-Seq result for both *C*. *moewusii *contigs and *C*. *reinhardtii *mRNA related to the structural and regulatory genes involved in starch catabolism, glycolysis, anaerobic fermentation.** Click here for file
