# Supplementary material for: De novo transcriptomic analysis of hydrogen production in the green alga Chlamydomonas moewusii through RNA-Seq
Source: Biotechnol Biofuels. 2013 Aug 23;6:118. doi: 10.1186/1754-6834-6-118 (PMC3846465; doi:10.1186/1754-6834-6-118)
Supplement: Additional file 1 — RNA-Seq data analysis 1 flowchart used in this study. [file 1754-6834-6-118-S1.doc]

**Additional File 1. RNA-Seq data analysis** 1 **flowchart used in this study.**
