# Supplementary material for: De novo transcriptomic analysis of hydrogen production in the green alga Chlamydomonas moewusii through RNA-Seq
Source: Biotechnol Biofuels. 2013 Aug 23;6:118. doi: 10.1186/1754-6834-6-118 (PMC3846465; doi:10.1186/1754-6834-6-118)
Supplement: Additional file 2 — General Information about the RNA-Seq Data. The dataset for 15 M-0502 was originally trimmed by sequencing facility and the data with red font were therefore not included for statistical calculation of the average (AVE) and standard deviation (SD). [file 1754-6834-6-118-S2.docx]

**Additional File 2. General Information about the RNA-Seq Data.** The dataset for 15M-0502 was originally trimmed by sequencing facility and the data with red font were therefore not included for statistical calculation of the average (AVE) and standard deviation (SD).

| **Sample ID** | **Time Point**  **(min)** | **Reads# (Million)** | **Sequence Length (bp)** | **Seq Duplication Level (%)** | **Length after Trim (bp)** | **Seq Duplication Level after Trim (%)** | **Length after trim and Duplicate Removal (bp)** | **Seq Duplication Level after Trim and Duplicate Removal (%)** |
| --- | --- | --- | --- | --- | --- | --- | --- | --- |
| **0M_0508** | **0** | **20** | **54** | **63** | **36** | **62** | **37** | **38** |
| **15M_0427** | **15** | **23** | **54** | **73** | **40** | **73** | **41** | **45** |
| **2H_0427** | **120** | **22** | **54** | **73** | **47** | **72** | **48** | **46** |
| **5H_0427** | **330** | **16** | **54** | **70** | **49** | **69** | **50** | **43** |
| **15M-0502** | **15** | **28** | **36** | **50** | **Original Trimmed** | **36** | **22** |  |
| **2H-0502** | **120** | **23** | **54** | **72** | **44** | **70** | **45** | **44** |
| **10H-0502** | **600** | **24** | **54** | **72** | **36** | **73** | **37** | **47** |
| **AVE** |  | **22.6** | **54** | **70.9** | **41.5** | **70.3** | **42.5** | **44.3** |
| **SD** |  | **3.2** | **0.0** | **3.3** | **5.2** | **3.7** | **5.2** | **2.9** |
