# Supplementary material for: De novo transcriptomic analysis of hydrogen production in the green alga Chlamydomonas moewusii through RNA-Seq
Source: Biotechnol Biofuels. 2013 Aug 23;6:118. doi: 10.1186/1754-6834-6-118 (PMC3846465; doi:10.1186/1754-6834-6-118)
Supplement: Additional file 3 — An example of improved RNA-Seq reads quality after data trimming. The ×6 axis on the FasrQC output graph of Per Base Sequence Quality shows the read position (bp), and Y-axis shows the quality scores with higher scores corresponding to better base calls. The Y-axis was divided into three regions with green background for very good quality calls (Quality score > =28), orange background for calls of reasonable quality (20 < =Quality score < 28), and red background for poor quality base calls (Quality score < 20). [file 1754-6834-6-118-S3.doc]

**Additional file 3**: An example of improved RNA-Seq reads quality after data trimming. The X6 axis on the FasrQC output graph of Per Base Sequence Quality shows the read position (bp), and Y-axis shows the quality scores with higher scores corresponding to better base calls. The Y-axis was divided into three regions with green background for very good quality calls (Quality score>=28), orange background for calls of reasonable quality (20<=Quality score<28), and red background for poor quality base calls (Quality score<20).
