# Supplementary material for: De novo transcriptomic analysis of hydrogen production in the green alga Chlamydomonas moewusii through RNA-Seq
Source: Biotechnol Biofuels. 2013 Aug 23;6:118. doi: 10.1186/1754-6834-6-118 (PMC3846465; doi:10.1186/1754-6834-6-118)
Supplement: Additional file 4 — The cumulative contig length with the increase of contig numbers (A) and statistical result of the length of assembled contigs using trimmed raw data with duplicate reads removed (B). X-axis is the number of contigs and Y-axis is the cumulative length of the contigs (kb) from contig_1 to contig_34136 (A). N: number of contigs used for statistical analysis. [file 1754-6834-6-118-S4.doc]

**Additional file 4**: The cumulative contig length with the increase of contig numbers (A) and statistical result of the length of assembled contigs using trimmed raw data with duplicate reads removed (B). X-axis is the number of contigs and Y-axis is the cumulative length of the contigs (kb) from contig_1 to contig_34136 (A). N: number of contigs used for statistical analysis.
