# Supplementary material for: De novo transcriptomic analysis of hydrogen production in the green alga Chlamydomonas moewusii through RNA-Seq
Source: Biotechnol Biofuels. 2013 Aug 23;6:118. doi: 10.1186/1754-6834-6-118 (PMC3846465; doi:10.1186/1754-6834-6-118)
Supplement: Additional file 6 — The relationship between C. moewusii transcripts generated through this study and the published C. moewusii mitochondrial genome sequence (NC_001872) (A), and BlastX results of two assembled contigs of contig_490 (B) and contig_1136 (C). 34,136 contigs were mapped against NC_001872 to investigate their relationship. Contigs with green color have same orientation as the reference and contigs with red color have reverse orientation as the reference (A). The graphic of B and C is an overview of NCBI NR database sequences aligned to the query sequence of contig_490 (the longest one, 15888-bp) and contig_1136 respectively. Alignments are color-coded by score, within one of five score ranges, the larger the number, the more conserved. Multiple alignments on the same database sequence are connected by a dashed line. [file 1754-6834-6-118-S6.doc]

**Additional file 6**: The relationship between *C. moewusii* transcripts generated through this study and the published *C. moewusii* mitochondrial genome sequence (NC_001872) (A), and BlastX results of two assembled contigs of contig_490 (B) and contig_1136 (C). 34,136 contigs were mapped against NC_001872 to investigate their relationship. Contigs with green color have same orientation as the reference and contigs with red color have reverse orientation as the reference (A). The graphic of B and C is an overview of NCBI NR database sequences aligned to the query sequence of contig_490 (the longest one, 15888-bp) and contig_1136 respectively. Alignments are color-coded by score, within one of five score ranges, the larger the number, the more conserved. Multiple alignments on the same database sequence are connected by a dashed line.
