# Supplementary material for: De novo transcriptomic analysis of hydrogen production in the green alga Chlamydomonas moewusii through RNA-Seq
Source: Biotechnol Biofuels. 2013 Aug 23;6:118. doi: 10.1186/1754-6834-6-118 (PMC3846465; doi:10.1186/1754-6834-6-118)
Supplement: Additional file 7 — Blast2GO results of combined graphs for the distributions of 34, 136 C. moewusii transcripts involved in different levels of Biological Processes (A), Molecular Function (B), and Cellular Components (C). And examples of enzymes identified by Blast2GO involved in pathways of One Carbon Pool by Folate (map00670) (D), C4-dicarboxylic Acid Cycle of Carbon Fixation in Photosynthetic Organism (map00710) (E). The enzymes in the pathways of D and E with colors highlighted are enzymes identified in C. moewusii, the ones without color highlighted are enzymes with no homologues identified in C. moewusii. [file 1754-6834-6-118-S7.doc]

**Additional file 7:** Blast2GO results of combined graphs for the distributions of 34, 136 *C. moewusii* transcripts involved in different levels of Biological Processes (A), Molecular Function (B), and Cellular Components (C). And examples of enzymes identified by Blast2GO involved in pathways of One Carbon Pool by Folate (map00670) (D), C4-dicarboxylic Acid Cycle of Carbon Fixation in Photosynthetic Organism (map00710) (E). The enzymes in the pathways of D and E with colors highlighted are enzymes identified in *C. moewusii,* the ones without color highlighted are enzymes with no homologues identified in *C. moewusii.*
