# Supplementary material for: De novo transcriptomic analysis of hydrogen production in the green alga Chlamydomonas moewusii through RNA-Seq
Source: Biotechnol Biofuels. 2013 Aug 23;6:118. doi: 10.1186/1754-6834-6-118 (PMC3846465; doi:10.1186/1754-6834-6-118)
Supplement: Additional file 8 — Parallel plot of RNA-Seq data distribution 1 before normalization (A) and Heat map and dendrogram (B), as well as parallel plot of RNA-Seq data distribution after normalization (C), and correlation scatterplots of the biological replicates at different phases (D4 F). (D: Phase I; E: Phase II; F: Phase III) using Pairwise method in JMP Genomics. The X-axis and Y-axis for A and C are the log2-based RPKM value and its corresponding density. [file 1754-6834-6-118-S8.doc]

**Additional file 8:** Parallel plot of RNA-Seq data distribution 1 before normalization (A) and Heat map and dendrogram (B), as well as parallel plot of RNA-Seq data distribution after normalization (C), and correlation scatterplots of the biological replicates at different phases (D4 F). (D: Phase I; E: Phase II; F: Phase III) using Pairwise method in JMP Genomics. The X-axis and Y-axis for A and C are the log2-based RPKM value and its corresponding density.
