# Supplementary material for: De novo transcriptomic analysis of hydrogen production in the green alga Chlamydomonas moewusii through RNA-Seq
Source: Biotechnol Biofuels. 2013 Aug 23;6:118. doi: 10.1186/1754-6834-6-118 (PMC3846465; doi:10.1186/1754-6834-6-118)
Supplement: Additional file 10 — Volcano plots of One-Way ANOVA analysis results (A: Phase I/Phase II; B: Phase II/Phase III; C: Phase I/Phase III) using JMP Genomics 6.0. The X-axis is the log2-based ratio and Y-axis is the value of –log10P-value. The horizontal dashed red line indicates the significance level with the values above the level are statistically significant. [file 1754-6834-6-118-S10.doc]

**Additional file 10**: Volcano plots of One-Way ANOVA analysis results (A: Phase I/Phase II; B: Phase II/Phase III; C: Phase I/Phase III) using JMP Genomics 6.0. The X-axis is the log2-based ratio and Y-axis is the value of –log10P-value. The horizontal dashed red line indicates the significance level with the values above the level are statistically significant.
