# Supplementary material for: De novo transcriptomic analysis of hydrogen production in the green alga Chlamydomonas moewusii through RNA-Seq
Source: Biotechnol Biofuels. 2013 Aug 23;6:118. doi: 10.1186/1754-6834-6-118 (PMC3846465; doi:10.1186/1754-6834-6-118)
Supplement: Additional file 12 — Examples of KEGG metabolic pathways that the 34,136 C. moewusii transcripts with hits to C. reinhardtii. 176 C. moewusii transcripts homologous to C. reinhardtii involved in Purine Metabolism (A), 125 C. moewusii transcripts homologous to C. reinhardtii involved in spliceosome (B), and 125 C. moewusii transcripts with homologous to C. reinhardtii involved in KEGG pathway of Carbon Fixation in Photosynthetic Organisms (C). The enzyme codes with red color indicate that homologues existing in C. moewusii. The cut-off E-value is 10-6 to identify C. moewusii homologues in C. reinhardtii. [file 1754-6834-6-118-S12.doc]

**Additional file 12:** Examples of KEGG metabolic pathways that the 34,136 *C. moewusii* transcripts with hits to *C. reinhardtii.* 176 *C. moewusii* transcripts homologous to *C. reinhardtii* involved in Purine Metabolism (A), 125 *C. moewusii* transcripts homologous to *C. reinhardtii* involved in spliceosome (B), and 125 *C. moewusii* transcripts with homologous to *C. reinhardtii* involved in KEGG pathway of Carbon Fixation in Photosynthetic Organisms (C). The enzyme codes with red color indicate that homologues existing in *C. moewusii*. The cut-off E-value is 10-6 to identify *C. moewusii* homologues in *C. reinhardtii.*
